# Capillary Electrophoresis Optimization for Metabolite Separation in *Hypogymnia physodes* Using DoE: Validation Across Lichen Species

**DOI:** 10.3390/ijms26104828

**Published:** 2025-05-18

**Authors:** Sławomir Dresler, Aneta Hałka-Grysińska, Izabela Baczewska, Hanna Wójciak, Barbara Hawrylak-Nowak, Jozef Kováčik, Olha Mykhailenko, Christian Zidorn, Joanna Sagan, Agnieszka Hanaka

**Affiliations:** 1 Department of Analytical Chemistry, Medical University of Lublin, Chodźki 4a, 20-093 Lublin, Poland; 2 Department of Plant Physiology and Biophysics, Institute of Biological Sciences, Maria Curie-Skłodowska University, Akademicka 19, 20-033 Lublin, Poland; 3 Department of Physical Chemistry, Medical University of Lublin, Chodźki 4a, 20-093 Lublin, Poland; 4 Department of Botany, Mycology and Ecology, Institute of Biological Sciences, Maria Curie-Skłodowska University, Akademicka 19, 20-033 Lublin, Poland; 5 Department of Botany and Plant Physiology, Faculty of Environmental Biology, University of Life Sciences in Lublin, Akademicka 15, 20-950 Lublin, Poland; 6 Department of Biology, University of Trnava, Priemyselná 4, 918 43 Trnava, Slovakia; 7 Department of Pharmaceutical Chemistry, National University of Pharmacy, 61168 Kharkiv, Ukraine; 8 School of Pharmacy, University College London, 29-39 Brunswick Square, London WC1N 1AX, UK; 9 Department of Pharmaceutical Biology, Kiel University, 24118 Kiel, Germany; 10 Division of Pharmaceutical Biotechnology, Department of Pharmaceutical, Biology and Biotechnology, Wroclaw Medical University, Borowska 211, 50-556 Wrocław, Poland

**Keywords:** lichen metabolites, phytochemical profiling, capillary electrophoresis, separation method optimization, cross-species validation, design of experiments (DoE)

## Abstract

Lichen-specific natural products exhibit a wide range of biological activities, which makes them potentially useful in the pharmaceutical, cosmetic, and nutritional industries. In the present study, a capillary electrophoresis method was developed and optimized for the separation of seven major metabolites, physodic acid, 3-hydroxyphysodic acid, atranorin, physodalic acid, chloroatranorin, salazinic acid, and protocetraric acid, found in *Hypogymnia physodes*. The optimization was performed using a design of experiments approach, focusing on four critical parameters: boric acid concentration, deoxycholic acid concentration, methanol content, and buffer pH. The overall separation efficiency was used as the response factor for optimization. The optimal separation conditions were achieved using a buffer composed of 60 mM boric acid, 70 mM deoxycholic acid, and 14% methanol at pH 9.6. The validated method was subsequently applied for the chemophenetic analysis of 28 lichen species belonging to the families Cladoniaceae, Parmeliaceae, Physciaceae, Ramalinaceae, and Teloschistaceae. In addition to the above-mentioned lichen compounds, the lichens examined showed the presence of evernic acid, usnic acid, and physicon. The developed CE method offers a reliable and efficient tool for the characterization of lichen metabolites, with potential applications in both botany and natural product research.

## 1. Introduction

Lichens represent a highly specialized group of organisms, consisting of obligate symbiotic associations between fungi (mycobionts) and algae and/or cyanobacteria (photobionts). One of their distinguishing features is the ability to synthesize unique specialized natural products, many of which are not found in other organisms and exhibit a wide range of biological activities [1]. These metabolites, predominantly polyphenolic in nature, confer antioxidant properties and have been reported to possess antibacterial, antiviral, anti-inflammatory, anticancer, and neuroprotective activities [2]. A comprehensive understanding of the chemical diversity and biological functions of lichen natural products, as well as the identification of new possibilities for their potential application, requires the development of robust qualitative and quantitative analytical methods. To date, chromatographic techniques, ranging from traditional thin-layer chromatography (TLC) [3] to more advanced high-performance liquid chromatography (HPLC) [4], have long been employed in the analysis of these compounds. More recently, liquid chromatography coupled with mass spectrometry detectors (LC-MS) have also been utilized to enhance sensitivity and selectivity [5]. In addition to chromatographic techniques, capillary electrophoresis (CE) represents a valuable and useful alternative for the analysis of plant-derived natural products [6]. Despite the fact that this technique offers numerous advantages, including relatively low sample and reagent consumption and versatile capabilities for the analysis of a wide range of analytes, it remains underutilized in the study of lichen metabolites profiling [7,8]. To the best of our knowledge, capillary zone electrophoresis (CZE) has previously been applied to the determination of usnic acid [8]. Meanwhile, micellar electrokinetic chromatography (MEKC) has also been optimized for the simultaneous determination of three metabolites in *Cladonia stellaris*—usnic acid, perlatolic acid, and atranorin [7].

The performance of electrophoretic separation is influenced by a wide range of experimental parameters, including applied voltage, separation temperature, and capillary properties. However, the most critical factor influencing resolution of the method is the composition and physicochemical properties of the background electrolyte (BGE). The primary parameters subject to optimization include the type and concentration of the buffer, its pH, and the presence of organic modifiers or solvents [9]. The most commonly used approach to optimizing electrophoretic conditions is the univariate method, in which a single parameter is varied within a defined range while all other factors are held constant. Although conceptually simple, this approach has two major limitations: (i) a comprehensive evaluation of all relevant variables requires a large number of individual experiments, making the process time and resource consuming; (ii) it does not take into account potential interactions between variables; if such interactions are present, the final conditions selected may differ significantly from the true optimum [10].

Taking into account the above limitations and considering the benefits of the design of experiments (DoE) approach, including the identification of significant factors and interactions as well as improved process understanding, the primary objective of this study was to develop and optimize a CE method for the separation of natural products in *Hypogymnia physodes*. We hypothesize that the application of DoE will allow a more effective and comprehensive optimization of CE separation parameters, ultimately improving separation resolution and analytical insight into the profiles of lichen extracts. To evaluate the overall performance of the optimized method, a novel index of overall separation efficiency was proposed. This index integrates balanced resolution coefficients of individual analytes, providing a holistic measure of separation quality. The optimized CE method was subsequently applied to the analysis of 28 lichen species. The resulting phytochemical profiles were compared and interpreted using chemometric methods, allowing for the assessment of species-specific metabolite patterns and potential chemotaxonomic relationships.

## 2. Results and Discussion

### 2.1. Phytochemical Characteristics of H. physodes

**Figure 1:**
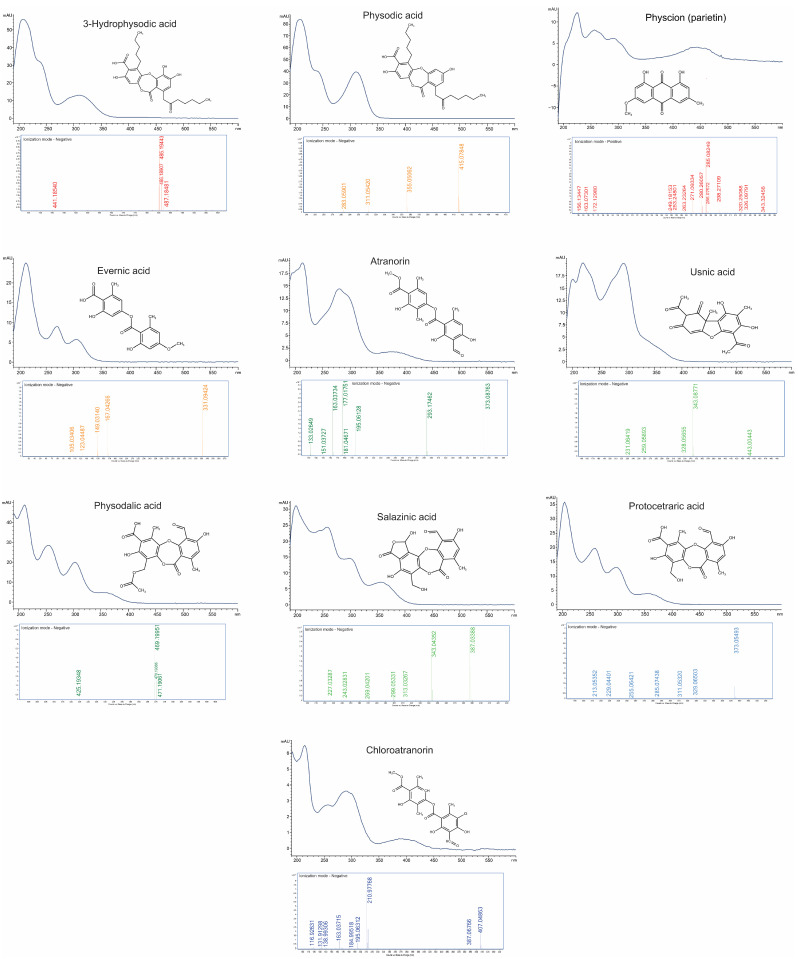
Combined presentation of capillary electrophoresis CE-UV-VIS spectra (190–600 nm), mass spectra (LC-TOF-MS), and chemical structures of ten selected lichen-derived compounds.

**Figure 2:**
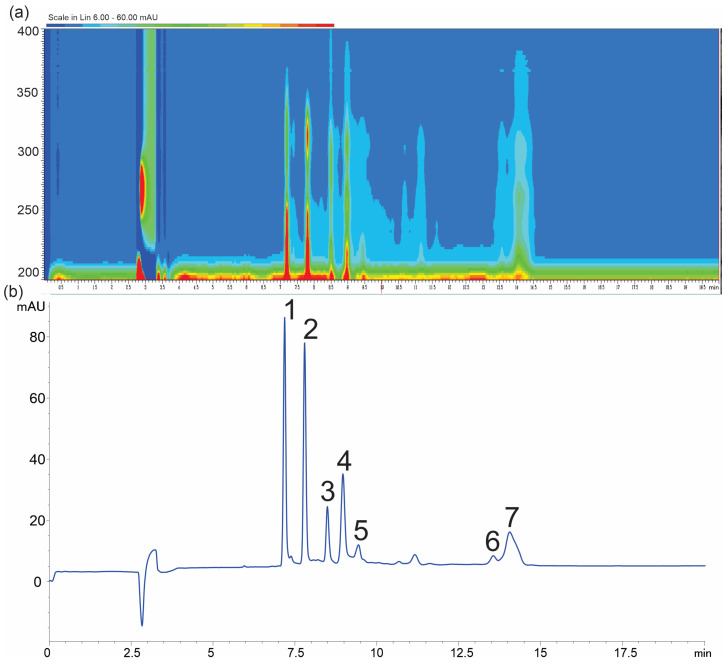
Electropherogram at 218 nm (**a**) and isoabsorbance plot of *H. physodes* extracts (**b**), (**1**) physodic acid, (**2**) 3-hydroxyphysodic acid, (**3**) atranorin, (**4**) physodalic acid, (**5**) chloroatranorin, (**6**) salazinic acid, and (**7**) protocetraric acid. Conditions: background electrolyte 60 mM boric acid, 70 mM deoxycholic acid concentration (DOC), and 14% MeOH at pH 9.6.

*Hypogymnia physodes* is a foliose lichen species belonging to the Parmeliaceae family. A distinctive feature of this species is its ability to accumulate significant amounts, up to 25%, of natural products belonging to various orcinol-type depsidones and depsides [11,12]. Previous reports indicate that the predominant compounds present in *H. physodes* include physodic acid, physodalic acid, 3-hydroxyphysodic acid, chloroatranorin, and atranorin [4,13]. Additional reports have also suggested the occurrence of protocetraric acid and salazinic acid in this species [14]. The identification of analytes was carried out based on the comparison of CE-UV-VIS spectra (with at least 95% similarity of the 192–600 nm spectrum) and migration times with those of reference standards. Additionally, the identity of the analytes was confirmed by comparative analysis of standards and extracts using liquid chromatography coupled with time-of-flight mass spectrometry (LC-TOF-MS) (Figure 1). According to the confidence levels for phytochemical identification [15], the compounds evaluated were assigned a confidence level of C, with the exception of chloroatranorin, whose identification was based solely on the literature data and tentative identification by LC-TOF analysis. In the present study, the presence of seven major lichen metabolites was confirmed in *H*. *physodes.* Among them, five compounds are classified as depsidones (physodic acid, physodalic acid, 3-hydroxyphysodic acid, salazinic acid, and protocetraric acid), while two compounds are classified as a depsides (atranorin and chloroatranorin) (Figure 2). These results are consistent with and extend previous chemical characterizations of the species [4,13,14].

### 2.2. Optimization of H. physodes Metabolites CE Separation

Design of experiments (DoE) is a useful statistical approach that facilitates the systematic investigation of complex processes through the controlled variation of multiple factors. Over the past few decades, DoE has been successfully applied to the optimization of several analytical separation techniques, including high-performance liquid chromatography (HPLC) and capillary electrophoresis (CE) [10,16]. Its application in analytical chemistry has led to significant improvements in the optimization and development of separation methods in terms of resolution, analysis time, and overall method robustness, demonstrating its value in the development of high-performance analytical protocols [16].

**Figure 3:**
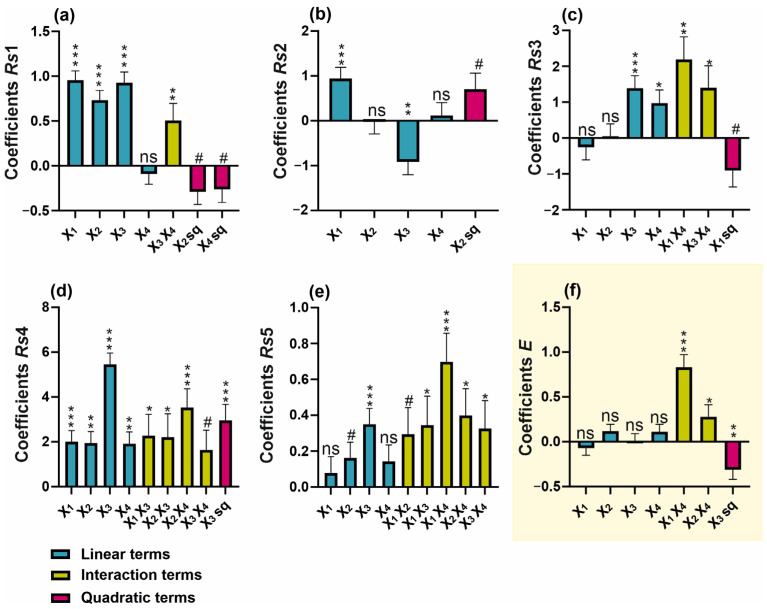
Regression coefficient plots of significant effects for the responses of (**a**) *Rs*1, (**b**) *Rs*2, (**c**) *Rs*3, (**d**) *Rs*4, (**e**) *Rs*5, and (**f**) overall separation efficiency (*E*). # *p* < 0.1; * *p* < 0.05; ** *p* < 0.01; *** *p* < 0.001; ns—not significant (*p* ≥ 0.1).

**Figure 4:**
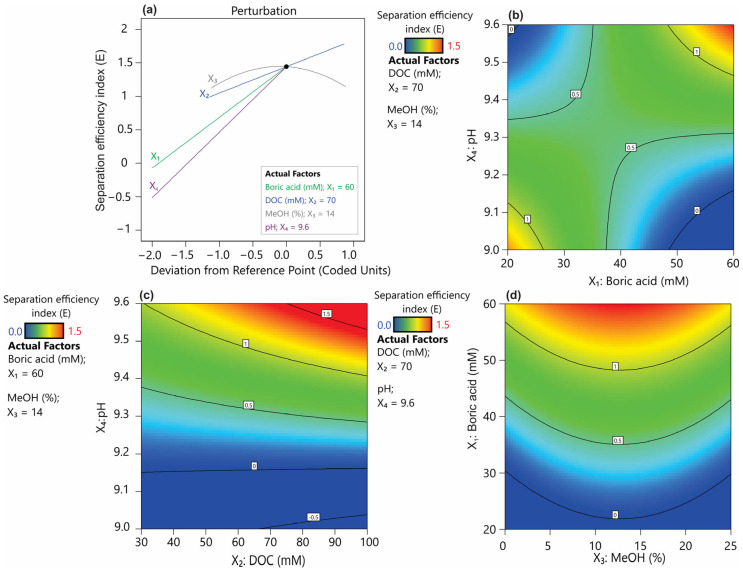
Piepel’s trace plot (**a**) and response surface plots showing the effect of buffer properties on the separation efficiency index (*E*): (**b**) pH and DOC, (**c**) pH and boric acid, and (**d**) boric acid and methanol.

In this study, we assumed that certain instrumental parameters, such as voltage and temperature, would not be subjected to optimization due to their predictable effects on CE performance [17]. Instead, their values were set to maximize the electroosmotic flow (EOF) and minimize overall analysis time, while ensuring a safe, low level of heat generation. Additionally, preliminary experiments indicated that a capillary with an internal diameter of 50 µm provided effective analyte separation while maintaining relatively high method sensitivity. While instrumental parameters can be somewhat predictable, particularly in terms of separation speed and reproducibility, buffer composition appears to be the key factor in determining resolution, selectivity, and efficiency. Four buffer-related variables were optimized to achieve the best separation of analytes: X_1_—boric acid concentration (20–60 mM), X_2_—deoxycholic acid concentration (DOC) (30–100 mM), X_3_—the presence of MeOH (0–25%), and X_4_—pH (9.0–9.6). Based on a series of experiments conducted according to the experimental design (Supplementary Table S1), polynomial regression models were developed for both individual resolution values and the overall separation efficiency index (*E*) (Table 1, Figure 3). Based on the obtained models, response surface projections were generated with respect to the most significant factors and the response expressed as the separation efficiency index (Figure 4). The models obtained showed a high degree of statistical significance and, with the exception of *Rs*4, no significant lack of fit. The determination coefficient (*R*^2^) for the developed models ranged from 0.52 to 0.92, while the predicted *R*^2^ values varied between 0.43 and 0.89, maintaining a reasonable consistency with the adjusted *R*^2^, as the difference remained below the threshold of 0.2 [18]. Furthermore, all models demonstrated a strong signal-to-noise ratio, confirmed by Adequate Precision (Adeq Precision) values exceeding the recommended minimum of four. Models were refined by removing terms with low statistical significance (*p* > 0.1) (Figure 3).

Boric acid-based buffers are among the most commonly used buffers in the CZE system, demonstrating high utility within the pH range of 8.1–10.1 and excellent transparency in the UV-VIS range [17]. The typical concentration of CE buffers ranges from 20 to 100 mM. Although higher buffer concentrations generally can enhance complexation with the analytes, leading to improved selectivity, they also lead to increased viscosity, reduced electroosmotic flow (EOF), and excessive heating, all of which can result in unstable separation [17]. The conducted studies indicated that increased boric acid concentration prolongs the migration time (Supplementary Figure S1a). It was also observed that this parameter has a certain linear significance in improving resolution (*Rs*1, *Rs*2, and *Rs*4, Figure 3a,b,d). Additionally, its quadratic term, which showed *p* < 0.1, was incorporated into the model for *Rs*3 (Figure 3c). Nevertheless, in the case of the resolution factors *Rs*3, *Rs*5, and the overall separation efficiency index (Figure 3c,e,f), the interaction between boric acid concentration and buffer pH proved to be significant. The pH of the background electrolyte (BGE) is a critical parameter influencing the ionization of both buffer components and the separated analytes. In the case of a borate buffer, pH determines the degree of boric acid dissociation, which indirectly affects the interaction between borate ions and analytes [17,19]. Undoubtedly, a key aspect of improving resolution in this method is achieving an optimal balance between boric acid dissociation, analyte complexation, and the impact of ionic strength on electrophoretic mobility. Based on the overall resolution index, it was found that the optimum setting ranges for these parameters would be a boric acid concentration of 60 mM and a pH of 9.6 (Figure 4a,b). It can be hypothesized that a high pH combined with a high boric acid concentration ensures a high level of borate ions in solution, thereby facilitating stronger interactions with hydroxyl-containing analytes. This, in turn, may enhance the separation efficiency.

Another effective method for improving separation efficiency in CE involves the addition of surfactants to the buffer, which can form micellar structures acting as a pseudostationary phase [20]. Deoxycholic acid (DOC) belongs to the class of bile acids and functions effectively as a surfactant in micellar electrokinetic chromatography (MEKC) [21]. Its concentration in the buffer is determined by the critical micelle concentration (CMC), reported to be approximately 5.4 mM in pure water at 25 °C. However, under electrophoretic conditions, the optimal concentration is estimated to be in the range of 20–75 mM [22]. An increase in DOC concentration has a significant positive effect on the resolution factors *Rs*1, *Rs*4, and *Rs*5, as well as prolonging the migration time of analytes. However, it should be noted that concentration of DOC closely interacts with the pH of the buffer, affecting *Rs*4, *Rs*5 and the electrophoretic efficiency factor (*E*) (Figure 4c). The interaction between these two parameters may be due to the effect of pH on the ionization level of DOC. In general, DOC as a bile salt derived from a weak acid, tends to form aggregates in neutral to alkaline conditions [22]. The models obtained showed that the optimal conditions for maximizing separation, both in terms of resolution and the efficiency factor, correspond to a DOC concentration of 70 mM combined with a buffer pH of 9.6.

Methanol was found to be a key factor influencing the separation efficiency of all analytes (Figure 3). Increasing the methanol concentration resulted in a pronounced prolongation of the retention time of analytes (Supplementary Figure S1c), a phenomenon consistent with previous findings [20,23]. It was pointed out that the use of organic modifiers, such as methanol, improves the solubility of analytes while also enhancing the separation efficiency of the MEKC method. This effect results from an increased difference between the migration time of non-interacting analytes and those that strongly bind to micelles [17]. In the present study, increasing methanol concentrations improved the coefficients *Rs*1, *Rs*3, *Rs*4, and *Rs*5; however, for physodic acid and atranorin (*Rs*2), high methanol concentrations limited their separation efficiency (Figure 4a,d). As a consequence of this heterogeneous effect of methanol on analyte resolution, the overall separation index (*E*) exhibited a quadratic function dependence on methanol concentration (Figure 3f). Therefore, the optimal methanol concentration resulting in a high E index was within the average range of the methanol concentrations tested (0–25%) and was determined to be 14%.

### 2.3. Validation of Method

Five concentration levels for each of the nine analytes were evaluated. Based on the acquired data, calibration curves were constructed by plotting corrected peak areas against the corresponding concentrations of each compound (Table 2). The developed method demonstrated satisfactory linearity, with correlation coefficients (*R*^2^) exceeding 0.994 for all compounds. The limit of detection (LOD), determined based on a signal-to-noise ratio (S/N) of three, ranged from 0.0807 to 0.4752 µg/mL. Accordingly, the limit of quantification (LOQ), calculated using a signal-to-noise ratio of 10, was established within the range of 0.5131 to 3.0183 µg/mL.

In the optimized method, the migration time of the analytes does not exceed 14.5 min (Table 3). Although CE is generally considered to be less reproducible than chromatographic techniques [24], the present study demonstrated high repeatability of migration times, with RSD% values ranging from 0.5 to 1.6%. Although the migration times in the developed method were relatively reproducible, the variations observed could still affect the accuracy of the quantitative analysis. Due to the differences in migration velocities of the compounds during CE separation, it was necessary to apply a correction to the peak areas (*CPA*) [25]. This correction was performed using the following equation to adjust the electropherograms accordingly [9]:

$$CPA=\frac{Peak\;area}{60\times MT}$$

The calculated values of RSD for the *CPA* for the individual compounds ranged from 1.9% to 9.7% (Table 3). The obtained repeatability values for both migration time and *CPA*, with an RSD below the commonly accepted threshold of 10%, indicate that the method demonstrates good precision [26].

### 2.4. Application of the Method for Analyzing Various Lichen Species

Three primary biosynthetic pathways of natural products in lichens can be distinguished: the shikimic acid pathway, the mevalonic acid pathway, and the acetate-polymalonate pathway. Among these, the acetate-polymalonate pathway plays a crucial role in the biosynthesis of the most common lichen acids. These metabolites include compounds based on phenolic structures composed of two or three orcinol or *β*-orcinol units [2]. This diverse group of natural products includes numerous compounds identified not only in *H. physodes*, but also in many other lichen species belonging to various families [27]. Using a developed and optimized CE method, acetone extracts from 28 lichen species were analyzed (Supplementary Table S2). In addition to the compounds previously identified in *H. physodes*, the method proved useful for the quantitative assessment of usnic acid, evernic acid, and physcion. A comparable approach was applied by Falk et al. [7], who used MEKC to separate evernic acid, usnic acid, and atranorin in *Cladina stellaris*, confirming the suitability of this technique for lichen metabolite analysis. However, it should be noted that the simultaneous presence of evernic acid and atranorin in the same sample observed in *E. prunastri* may pose a challenge due to their similar migration times. To improve the resolution between these two compounds, a modified buffer composition was employed: 40 mM boric acid, 100 mM deoxycholic acid (DOC), and 12.5% methanol at pH 9.6. The identity of all metabolites (with the exception of chloroatranorin) was confirmed by comparing their migration times and UV-VIS spectra (190–600 nm, at least 95% of similarity) with those of standards obtained commercially or isolated via chromatographic methods. Furthermore, metabolite identities were validated using mass spectrometry (Figure 1).

Although the developed method is applicable for the analysis of a wide range of lichen species, its sensitivity is fundamentally limited by the short effective optical path length. For a 50 µm i.d. capillary, this path length is approximately 32 µm, which markedly limits the detection capability of CE when compared to standard HPLC systems equipped with conventional UV-VIS detectors [20]. This limitation is reflected in the LOD and LOQ values (Table 2), which are substantially higher than those typically achieved by chromatographic techniques. For instance, in the case of usnic acid, the sensitivity of CE-UV-VIS is approximately five times lower than that of HPLC-UV-VIS [28]. However, it is worth noting that certain HPLC-based methods have demonstrated sensitivity levels for detected lichen metabolites that are comparable to those achieved with the CE-UV-VIS method described in this study [29,30]. This observation may be partially due to the use of different detection wavelengths. In our method, the measurement was performed at 218 nm, a wavelength at which lichen metabolites exhibit strong absorbance. In contrast, HPLC methods often employ longer wavelengths, which, although beneficial for selectivity, may result in lower signal intensity [29,30]. Nonetheless, this is effectively compensated by the longer optical path length in HPLC detectors compared to those used in capillary electrophoresis, ultimately yielding similar LOD values. Despite the fact that there are several strategies to increase the sensitivity of the CE method, the inherently low detection sensitivity of UV-VIS-based CE remains one of its major drawbacks [31].

**Figure 5:**
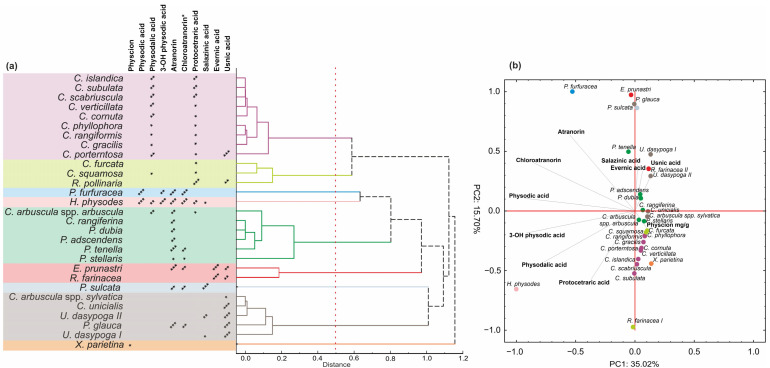
Chemotaxonomic relationships among lichen species based on secondary metabolite profiles. (**a**) Hierarchical cluster analysis (HCA) of species based on presence/absence of major lichen substances. (**b**) Principal component analysis (PCA) biplot showing distribution of species in relation to key natural products. Asterisks indicate approximate compound content: * ≤2 mg/g DW, ** = 2–10 mg/g DW, *** >10 mg/g DW. * The content of chloroatranorin was expressed as a relative quantity.

Given the inherently lower sensitivity of the CE method, it is important to be aware of both its analytical limitations and the fact that, in the described raw materials, only metabolite groups present in quantities sufficient for identification at the UV-VIS spectral level could be identified. Therefore, despite previous reports indicating the presence of atranorin, usnic acid, and vulpinic acid in *Xanthoria parietina* [32], their concentrations were probably too low to be detected using the CE method. Nevertheless, the CE-based analysis and identification of dominant groups of constituents present in the raw material may be valuable both from the perspective of material evaluation and chemometric classification. Moreover, despite its sensitivity limitations, CE provides several important advantages, including high separation efficiency, low sample and solvent consumption, and flexible buffer design. These features support its application in phytochemical studies of lichens as a sustainable and efficient analytical alternative to chromatographic techniques [33]. In the present study, a classification of the samples was carried out, resulting in the identification of nine homogeneous lichen groups (Figure 5a). The first two groups comprised species from the genus Cladonia, as well as *Cetraria islandica*, and *Ramalina farinacea*. The presence of protocetraric acid characterized these species and, except *Cladonia furcata* and *R. farinacea*, also physodalic acid. In the PCA analysis, these taxa were strongly influenced by the second principal component (PC2), which was associated with the presence of protocetraric acid (Figure 5b).

Another major group of lichens included species characterized by the presence of atranorin. This group was primarily composed of representatives of the genus *Physcia*, as well as *C. arbuscula* subsp. *arbuscula* and *C. rangiferina*. Although atranorin was also detected in other species, the occurrence of additional compounds influenced their classification into separate chemometric groups. For example, the presence of evernic acid defined a distinct group comprising *Evernia prunastri* and *Ramalina pollinaria*. Similarly, the presence of usnic acid defined a group consisting of five individuals including *C. arbuscula* subsp. *sylvatica*, *C. unicialis*, *Usnea dasypoga*, and *Platismatia glauca*.

A notably high content of salazinic acid distinguished *Parmelia sulcata*, while the occurrence of physcion was characteristic of *X. parietina*. The final two distinct groups were marked by both a generally high total content of lichen acids and the presence of at least four different compounds; these were *Pseudevernia furfuracea* and *H. physodes*. These two groups were clearly separated from the remaining studied species along the PC1 axes. In particular, *H. physodes* was strongly influenced by PC1, which accounted for 35% of the total variance and was primarily associated with the presence of physodic acid, 3-hydroxyphysodic acid, physodalic acid, chloroatranorin, protocetraric acid, and atranorin. Similarly, *P. furfuracea* was located on the right side of the PC1 axis. However, in this case, the extract was also strongly influenced by PC2, which was determined by the absence of protocetraric acid and physodalic acid in the extract (Figure 5b).

## 3. Materials and Methods

### 3.1. Chemicals and Reagends

The background electrolytes (BGE) were prepared using boric acid (>99.5%), sodium deoxycholate (DOC), methanol (MeOH, 99.9%, gradient grade), and 1 M NaOH for pH adjustment from Sigma-Aldrich (Merck KGaA, Darmstadt, Germany). Atranorin (>95%), salazinic acid (>95%), and protocetraric acid (>95%) were acquired from Cayman Chemical Company (Ann Arbor, MI, USA). Usnic acid (>98%) and physcion (analytical standard) were purchased from Sigma-Aldrich (Merck KGaA, Darmstadt, Germany). 3-Hydroxyphysodic acid, physodalic acid, and physodic acid were isolated from *Hypogymnia physodes* according to a previously described method [4]. The acetone (>99.5) used for preparing the extracts was obtained from Sigma-Aldrich (Merck KGaA, Darmstadt, Germany).

### 3.2. Lichen Material and Extraction Protocol

The optimization procedure for the separation of lichen acids using capillary electrophoresis was conducted with an acetone extract of *Hypogymnia physodes* (Parmeliaceae). Additionally, the method was applied to the analysis of 27 other lichen species from five families: *Cladonia arbuscula* subsp. *arbuscula*, *Cladonia arbuscula* subsp. *sylvatica*, *Cladonia cornuta*, *Cladonia furcata*, *Cladonia gracilis*, *Cladonia phyllophora*, *Cladonia rangiferina*, *Cladonia rangiformis*, *Cladonia scabriuscula*, *Cladonia squamosa*, *Cladonia subulata*, *Cladonia unicialis*, *Cladonia verticillata* (Cladoniaceae), *Cetraria islandica*, *Evernia prunastri*, *Parmelia sulcata*, *Platismatia glauca*, *Pseudevernia furfuracea*, *Usnea dasypoga* (Parmeliaceae), *Physcia adscendens*, *Physcia dubia*, *Physcia stellaris*, *Physcia tenella* (Physciaceae), *Ramalina farinacea*, *Ramalina pollinaria* (Ramalinaceae), and *Xanthoria parietina* (Teloschistaceae). In the case of *Usnea dasypoga*, specimens collected from two different locations exhibited certain distinct anatomical differences, which, however, were insufficient to definitively determine subspecific affiliation. As a result, only two separate populations were distinguished. The species were collected in the Lublin Voivodeship, Poland, during the autumn of 2024. Species identification was confirmed by lichenologist Dr. Hanna Wójciak. The collected samples were air-dried at room temperature and stored at 4 °C until extract preparation. The extraction procedure was adapted from [12] with minor modifications. Briefly, 50 mg of raw material was finely homogenized in a mortar with 2 mL of acetone at ambient temperature. The resulting suspension underwent ultrasound-assisted extraction at 35 kHz for 20 min within a temperature range of 30–35 °C, using a Sonorex RK 512H ultrasonic bath (Bandelin, Berlin, Germany). Following sonication, the extract was centrifuged at 10,000× *g* for 5 min, and the supernatant was transferred to a 5 mL tube. The remaining precipitate was re-extracted twice with fresh acetone portions. The pooled extracts were adjusted to a final volume of 5 mL and subsequently analyzed for lichen metabolites.

### 3.3. Equipment and CE Separation Conditions

Capillary electrophoresis (CE) analyses were conducted using an Agilent 7100 system, which featured a diode array spectrophotometric detector operating within the 190–600 nm range (Agilent Technologies, Santa Clara, CA, USA). Separations were performed with 50 μm i.d. capillaries (Agilent Technologies, Santa Clara, CA, USA) that had a total length of 64.5 cm, including an effective length of 56 cm. Before each sample injection, the capillary was rinsed sequentially with 0.1 M NaOH under approximately 1 bar pressure for 10 min, followed by deionized water for 10 min, and finally with the background electrolyte (BGE) for another 10 min. Samples were introduced by applying a pressure of 50 mbar for 4.0 s, followed by a 4.0 s BGE flush at the same pressure. The electrophoretic separations were carried out at 30 kV with a capillary temperature maintained at 27 °C.

### 3.4. Metabolite Identification and Validation of the Method

The identification of lichen-derived metabolites was performed in a multistep approach based on the comparison of CE-UV-VIS spectra and analyte migration times with those of authentic standards. In addition, analyte identity was further verified through parallel analyses using liquid chromatography (Ultra-High Performance Liquid Chromatography Infinity Series II, Agilent Technology, Santa Clara, CA, USA) coupled with time-of-flight mass spectrometry (Agilent 6224 ESI/TOF, Agilent Technologies, Santa Clara, CA, USA). This procedure was carried out in accordance with the method described by Hanaka et al. [34]. In the case of chloroatranorin, its identification was based on cross-comparison of different lichen extracts analyzed using both CE and LC-TOF-MS and was confirmed based on accurate mass evaluation. With the exception of chloroatranorin, whose identification was based solely on the literature data and tentative identification by LC-TOF analysis, the evaluated compounds were assigned a confidence level of C, according to the proposed confidence levels for phytochemical identification [15]. Absolute concentrations of the analytes (with the exception of chloroatranorin) were determined using calibration curves constructed from five-point dilution series (*n* = 5), with detection based on absorbance at 218 nm. Additionally, method validation was performed by evaluating both repeatability and precision (*n* = 5). The limits of detection (LOD) and quantification (LOQ) were calculated based on a signal-to-noise ratio of 3 and 10, respectively, using visual assessment of signal-to-noise levels.

### 3.5. Optimization of Separation Efficiency Using the D-Optimal Design

The D-optimal design was employed to evaluate the influence of tour independent numerical factors on the separation process: X_1_—boric acid concentration in BGE (mM); X_2_—DOC concentration in BGE (mM); X_3_—MeOH concentration in BGE (%); X_4_—pH of BGE. Polynomial models were constructed separately for each critical pair of peaks that exhibited a tendency toward co-elution. *Rs*1 corresponded to the peak of 3-hydroxyphysodic acid, *Rs*2 to atranorin, *Rs*3 to physodalic acid, *Rs*4 to chloroatranorin, and *Rs*5 referred to the separation of salazinic acid and protocetraric acid. Additionally, an overall separation efficiency index (*E*) was calculated using Equation (1)

(1) $$E=\bar{R_{s}\;}\;\cdot \;P$$

The overall separation efficiency index (*E*) ranges from 0 (no separation) to 1.5 (good separation). The index integrates the average resolution of all separated components, with two main considerations: first, resolution values above 1.5 will be limited to 1.5 in order to avoid overestimating the efficiency; second, resolution values below 1 will be penalized by a quadratic penalty function that compensates for insufficient separation, as described in Equation (2):

(2) $$P=\sum \frac{R_{sp}^{2}}{m^{1.25}}$$

However, the quadratic penalty function is applied only when the dataset *Rs* contains values below 1. In cases where all values are greater than or equal to 1, the value of *E* is equal to the mean of the *Rsi* values. This approach ensures that the overall efficiency index reflects both the completeness of separation and the presence of poorly resolved peaks. The extended formula for the overall separation efficiency (*E*) is given in Equation (3).

(3) $$E=\left(\frac{\sum_{i=1}^{n}R_{s,i})}{n}\right)\cdot \left(\frac{\sum_{j=1}^{m}R_{sp,j}^{2}}{m^{1.25}}\right)$$

$\bar{Rs}$—the average of all *Rsi* values

*R_s,i_*—represents all resolution values, where each *Rs* is limited to a maximum of 1.5

*R_sp,j_*—represents resolution values where *Rs* < 1.0

*P*—quadratic penalty function for *Rs* < 1

*n*—total number of *Rsi* values

*m*—number of *Rsp* values below 1.0

Based on the obtained response data, including the separation efficiency index, resolution coefficients, and migration time, polynomial models were developed and validated by assessing their significance using ANOVA and evaluating the normal distribution of residuals with the Shapiro–Wilk test (*p* < 0.05). Additionally, the models’ adequacy was verified by calculating fit statistics, including *R*^2^, adjusted *R*^2^, predicted *R*^2^, and adequate precision. In the final stage, numerical optimization of the method was carried out based on the maximization of the separation efficiency index, assuming an optimal separation time of up to 15 min in order to reduce the overall analysis time.

### 3.6. Classification Analysis

The chemophenetic [35] relationships among the species were illustrated using a dendrogram generated through hierarchical clustering based on a Pearson correlation distance matrix. In addition, principal component analysis (PCA) was conducted to explore patterns in the concentrations of lichen metabolites. All calculations were performed using Statistica software, version 6.1 (StatSoft, Inc., 2004, Tulsa, OK, USA).

## 4. Conclusions

This study shows that a design of experiments approach can be used to develop and optimize a capillary electrophoretic method for the separation of key natural products in *H. physodes*. The proposed index of effective separation allowed a generalized evaluation of the resolution efficiency of the method when analyzing multiple analytes simultaneously, providing a valuable metric for the development of predictive models within the DoE framework. Optimal separation conditions were achieved using a buffer consisting of 60 mM boric acid, 70 mM deoxycholic acid (DOC), 14% methanol, and pH 9.6, allowing effective resolution of all seven major metabolites present in *H. physodes*. Although organic solvents such as methanol are known to increase the critical micelle concentration (CMC) of surfactants, the DOC concentration applied here exceeds typical CMC thresholds, and its rigid steroidal structure likely contributed to maintaining micellar stability and separation efficiency under the chosen conditions. The method was successfully applied to the chemotaxonomic comparison of lichen species from four different genera. Buffer adjustment to improve separation of evernic acid and atranorin was required only for *E. prunastri* due to their similar migration times. The results highlight the potential of capillary electrophoresis as a powerful analytical tool in lichenological research. This has implications for both species classification and the study of biologically active natural products. In future applications, the incorporation of on-line sample preconcentration strategies such as stacking could be considered to improve detection sensitivity, particularly for low-abundance compounds.

## Figures and Tables

**Table 1:** Statistical summary of regression models.

Response		*p*-Value	*R* ^2^	Adj. *R*^2^	Pred. *R*^2^	Adeq Prec.
*Rs*1	Model	<0.0001	0.902	0.874	0.834	19.603
	Lack of fit	0.3151				
*Rs*2	Model	0.0008	0.516	0.423	0.296	8.834
	Lack of fit	0.7400				
*Rs*3	Model	<0.0001	0.673	0.588	0.465	10.375
	Lack of fit	0.0520				
*Rs*4	Model	<0.0001	0.922	0.892	0.846	19.957
	Lack of fit	0.041				
*Rs*5	Model	<0.0001	0.843	0.787	0.684	16.981
	Lack of fit	0.438				
*E*	Model	<0.0001	0.731	0.664	0.562	13.371
	Lack of fit	0.733				

**Table 2:** Linear regression results, LOD, and LOQ of the investigated compounds.

Compound	Calibration Curve	Correlation Coefficient	LOD (µg/mL)	LOQ (µg/mL)
3-Hydroxyphysodic acid	y = 1.466 x + 0.03137	0.998	0.2408	1.530
Physodic acid	y = 2.001 x − 0.06959	0.998	0.1865	1.184
Physcion	y = 2.690 x − 0.00064	0.995	0.1384	0.879
Evernic acid	y = 2.146 x − 0.02295	0.997	0.1597	1.014
Atranorin	y = 2.015 x + 0.00373	0.999	0.1775	1.127
Usnic acid	y = 2.210 x − 0.0423	0.998	0.2019	1.282
Physodalic acid	y = 0.689x + 0.0254	0.998	0.4752	3.018
Salazinic acid	y = 1.251 x + 0.0034	0.994	0.2514	1.597
Protocetraric acid	y = 0.554 x + 0.0610	0.996	0.0807	0.513

**Table 3:** Average migration time (*MT*), standard deviation (SD), and relative standard deviation (RSD) of migration time and corrected peak area (*CPA*) (*n* = 5).

Compound	*MT* (min)	*MT*_SD_	*MT*_RSD(%)_	*CPA*_SD_	*CPA*_RSD(%)_
3-Hydroxyphysodic acid	7.50	0.056	0.747	0.012	7.179
Physodic acid	8.17	0.074	0.902	0.019	9.730
Physcion	8.24	0.045	0.549	0.010	4.547
Evernic acid	8.68	0.132	1.523	0.003	1.882
Atranorin	8.94	0.102	1.144	0.006	8.152
Usnic acid	9.01	0.148	1.611	0.008	2.996
Physodalic acid	9.34	0.075	0.806	0.010	7.237
Salazinic acid	13.91	0.119	0.857	0.001	3.969
Protocetraric acid	14.59	0.136	0.934	0.011	4.883

## Data Availability

The data associated with this research can be accessed at https://doi.org/10.5281/zenodo.15449430.

## Supplementary Materials

The following supporting information can be downloaded at: https://www.mdpi.com/article/10.3390/ijms26104828/s1.
