# Supplementary material for: Capillary Electrophoresis Optimization for Metabolite Separation in Hypogymnia physodes Using DoE: Validation Across Lichen Species
Source: Int J Mol Sci. 2025 May 18;26(10):4828. doi: 10.3390/ijms26104828 (PMC12112299; doi:10.3390/ijms26104828)
Supplement: Supplementary file 1 [file ijms-26-04828-s001.zip › ijms-3634851-supplementary.pdf]

SUPPLEMENTARY MATERIALS

Capillary Electrophoresis Optimization for Metabolite Separation in *Hypogymnia physodes* Using DoE: Validation Across Lichen Species

Sławomir Dresler [1,2,*], Aneta Hałka-Grysińska [3], Izabela Baczewska [1], Hanna Wójciak [4], Barbara Hawrylak-Nowak [5], Jozef Kováčik [6], Olha Mykhailenko [7,8,9], Christian Zidorn [9,10], Joanna Sagan [2] and Agnieszka Hanaka [2,*]

[1] Department of Analytical Chemistry, Medical University of Lublin, Chodźki 4a, 20-093 Lublin, Poland
[2] Department of Plant Physiology and Biophysics, Institute of Biological Sciences, Maria Curie-Skłodowska University, Akademicka 19, 20-033 Lublin, Poland
[3] Department of Physical Chemistry, Medical University of Lublin, Chodźki 4a, 20-093 Lublin, Poland
[4] Department of Botany, Mycology and Ecology, Institute of Biological Sciences, Maria Curie-Skłodowska University, Akademicka 19, 20-033 Lublin, Poland
[5] Department of Botany and Plant Physiology, Faculty of Environmental Biology, University of Life Sciences in Lublin, Akademicka 15, 20-950 Lublin, Poland
[6] Department of Biology, University of Trnava, Priemyselná 4, 918 43 Trnava, Slovakia
[7] Department of Pharmaceutical Chemistry, National University of Pharmacy, 61168 Kharkiv, Ukraine
[8] School of Pharmacy, University College London, 29-39 Brunswick Square, London WC1N 1AX, UK
[9] Department of Pharmaceutical Biology, Kiel University, 24118 Kiel, Germany
[10] Division of Pharmaceutical Biotechnology, Department of Pharmaceutical, Biology and Biotechnology, Wroclaw Medical University, Borowska 211, 50-556 Wrocław, Poland
* Correspondence: slawomir.dresler@umlub.pl (S.D.); agnieszka.hanaka@poczta.umcs.lublin.pl (A.H.)

Table S1. Response function results of the response surface analysis.

X1	X2	X3	X4	Response
Boric acid (mM)	DOC (mM)	MeOH (%)	pH	Separation efficiency index (*E*)
20	30	12.5	9.3	0.74
20	65	25	9.3	0.44
20	65	12.5	9	1.43
20	65	12.5	9.6	0.20
20	65	12.5	9.33	0.23
20	65	10	9.3	0.43
20	65	25	9.3	0.08
20	100	12.5	9.3	1.04
40	30	0	9.3	0.00
40	30	12.5	9	0.28
40	30	12.5	9.6	0.00
40	30	12.5	9.3	0.33
40	65	0	9.6	0.15
40	65	12.5	9.3	0.75
40	65	25	9.6	0.00
40	65	25	9	0.06

40	65	12.5	9.3	0.00
40	65	0	9	0.33
40	65	0	9.3	0.21
40	100	12.5	9.6	1.10
40	100	0	9.3	0.16
40	100	12.5	9	0.05
40	100	12.5	9.3	0.23
60	30	12.5	9.3	0.31
60	65	0	9.3	0.36
60	65	12.5	9.6	1.41
60	65	25	9.3	0.00
60	100	12.5	9.3	1.12
60	65	12.5	9.3	0.20
40	65	25	9.3	0.27
40	65	18.75	9.3	1.02
60	100	18.75	9.6	1.50
40	30	25	9.3	0.45
60	65	18.75	9.6	1.50
60	65	18.75	9.6	1.50

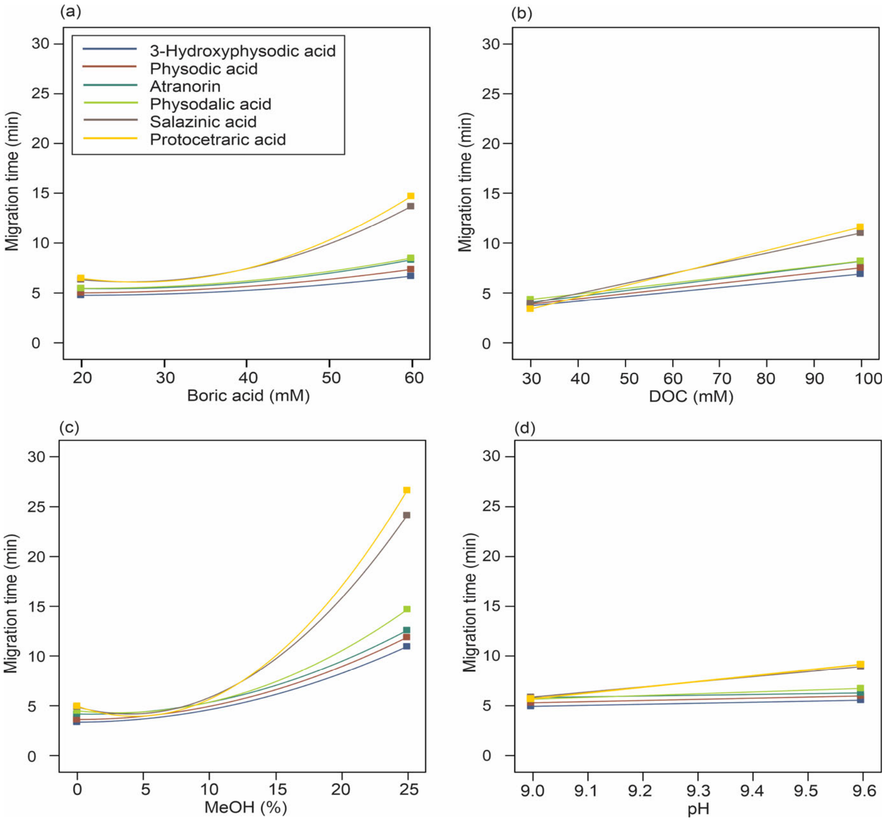

Figure S1. The effect of (a) boric acid concentration, (**b**) DOC, (**c**) MeOH, and (**d**) buffer pH on the migration time of individual analytes.

SUPPLEMENTARY MATERIALS

Table S2. Mean concentration (±SD) of predominant metabolites in the studied lichen species (*n*=3).

Species	Usnic acid	3-OH physodic acid	Physodic acid	Physodalic acid	Atranorine	Chloroatranorine*	Salazinic acid	Protocetraric acid	Evernic acid	Physcion
Cetraria islandica				6.58 ±8.19				2.96 ±1.76		
C. arbuscula spp. arbuscula				2.12 ±2.67	6.27 ±4.56			1.42 ±0.59		
C. arbuscula spp. sylvatica	1.28 ±4.78									
Cladonia cornuta				3.35 ±3.36				1.58 ±0.61		
Cladonia furcata								0.39 ±0.29		
Cladonia gracilis				1.89 ±1.17				1.24 ±0.26		
Cladonia phyllophora				0.79 ±0.36				0.67 ±0.92		
Cladonia porterntosa	2.22 ±0.19			4.65 ±0.67				1.72 ±0.13		
Cladonia rangiferina					4.99 ±0.68					
Cladonia rangiformis				0.95 ±1.64				0.78 ±0.21		
Cladonia scabriuscula				7.58 ±1.42				2.35 ±0.13		
Cladonia squamosa				0.17 ±0.29				0.46 ±0.26		
Cladonia subulata				9.56 ±6.86				2.94 ±1.89		
Cladonia unicialis	14.48 ±6.27									
Cladonia verticillata				4.36 ±1.00				1.57 ±0.60		
Evernia prunastri	7.72 ±0.46				18.13 ±2.32	4.21 ±1.84			28.26 ±2.23	
Hypogymnia physodes		41.45 ±8.70	23.78 ±3.67	3.59 ±7.26	13.78 ±6.63	13.98 ±2.43	1.53 0.25	8.83 ±5.99		
Parmelia sulcata					7.54 ±2.97	5.88 ±1.43	53.93 18.77			
Physcia adscendens					9.45 ±2.21					
Physcia dubia					8.17 ±2.87					
Physcia stellaris					1.19 ±0.28	1.54 ±0.38				
Physcia tenella					15.95 ±0.55	5.19 ±0.72				
Platismatia glauca	37.85 ±8.94				18.32 ±25.69	3.97 ±0.63				
Pseudevernia furfuracea		6.95 2.20	24.92 ±2.44		15.66 ±17.74	21.98 ±8.75				
Ramalina pollinariaI	8.73 ±4.36							17.67 ±11.55		

Ramalina farinacea	3.64 ±0.83		38.32 ±6.59	
Usnea dasypoga	37.54 ±12.59	2.47 ±7.81		
Usnea dasypoga 2	37.47 ±11.46	5.23 ±0.35		
Xanthoria parietina				1.88 ±1.42

*Relative content (per g dry weight) determined from corrected peak area
